# EDBD—3,6-Epidioxy-1,10-Bisaboladiene—An Endoperoxide Sesquiterpene Obtained from *Drimys brasiliensis* (Winteraceae) Exhibited Potent Preclinical Efficacy against *Schistosoma mansoni* Infection

**DOI:** 10.3390/antibiotics13080779

**Published:** 2024-08-18

**Authors:** Eric Umehara, Thainá R. Teixeira, Rayssa A. Cajás, Monique C. Amaro, Josué de Moraes, João Henrique G. Lago

**Affiliations:** 1 Centro de Ciências Naturais e Humanas, Universidade Federal do ABC, Santo André 09280-560, SP, Brazil; ericumehara@hotmail.com; 2 Centro de Pesquisas de Doenças Negligenciadas, Universidade Guarulhos, Guarulhos 07023-070, SP, Brazil; thainanpdn@gmail.com (T.R.T.); moniquecamaro2004@gmail.com (M.C.A.); 3 Núcleo de Pesquisas em Doenças Negligenciadas, Instituto Científico e Tecnológico, Universidade Brasil, São Paulo 08230-030, SP, Brazil

**Keywords:** antischistosomal activity, 3,6-epidioxy-1,10-bisaboladiene, *Drimys brasiliensis*, antiparasitic properties, schistosomiasis

## Abstract

Schistosomiasis, a neglected tropical disease impacting over 250 million individuals globally, remains a major public health challenge due to its prevalence and significant impact on affected communities. Praziquantel, the sole available treatment, highlights the urgency of the need for novel anthelmintic agents to achieve the World Health Organization (WHO) goal of schistosomiasis elimination. Previous studies reported the promising antiparasitic activity of different terpenoids against *Schistosoma mansoni* Sambon (Diplostomida: Schistosomatidae). In the present work, the hexane extract from branches of *Drimys brasiliensis* afforded a diastereomeric mixture of endoperoxide sesquiterpenes, including 3,6-epidioxy-bisabola-1,10-diene (EDBD). This compound was evaluated in vitro and in vivo against *S. mansoni.* EDBD exhibited a significant reduction in *S. mansoni* viability in vitro, with an effective concentration (EC_50_) value of 4.1 µM. Additionally, EDBD demonstrated no toxicity to mammalian cells. In silico analysis predicted good drug-likeness properties, adhering to pharmaceutical industry standards, including favorable ADME profiles. Furthermore, oral treatment of *S. mansoni*-infected mice with EDBD (400 mg/kg) resulted in a remarkable egg burden reduction (98% and 99% in tissues and feces, respectively) surpassing praziquantel’s efficacy. These findings suggest the promising potential of EDBD as a lead molecule for developing a novel schistosomiasis treatment.

## 1. Introduction

Worm parasites contribute significantly to the global disease burden, affecting nearly 1.8 billion people worldwide, particularly in regions with inadequate healthcare and socioeconomic conditions. Among these parasites, the blood fluke of the genus *Schistosoma* is noteworthy for its responsibility in nearly 240 million cases of schistosomiasis worldwide, posing a risk of infection to approximately 10% of the human population [1]. Chronic infection can lead to significant morbidity, with an estimated 1.5 million disability-adjusted life years (DALYs) lost due to schistosomiasis [2]. *Schistosoma mansoni* Sambon (Diplostomida: Schistosomatidae) adult worms reside within the human portal vasculature, often for years. They release eggs that can travel in two paths: either passing through the intestinal wall and exiting in the feces or migrating to the liver. In the liver, the eggs trigger an immune response, leading to the formation of granulomas and peri-portal fibrosis [3].

The World Health Organization’s (WHO) neglected tropical diseases roadmap 2021–2030 aims to eliminate schistosomiasis as a public health issue and interrupt its transmission in selected countries [4]. However, the current strategy heavily relies on praziquantel (PZQ), leading to concerns about drug resistance and efficacy due to its widespread use [5]. Given the limitations of PZQ, there is a pressing need for alternative treatments [6]. Natural products are pivotal in drug discovery for treating infectious diseases due to their unique characteristics compared to synthetic molecules [7]. Plants of the genus *Drimys* (Winteraceae), including *D. brasiliensis*, are rich sources of specialized metabolites, such as sesquiterpenes with antifungal [8], anti-*Leishmania* [9], antimalarial [9], and anti-inflammatory activity [10] as well as alkaloids with antinociceptive effects [11]. *D. brasiliensis*, found across the Brazilian Atlantic Forest, Cerrado, and Caatinga biomes, holds particular promise in this regard [12]. While previous research has explored sesquiterpenes from *D. brasiliensis* for anti-*Trypanosoma cruzi* activity [13], their potential against *Schistosoma* remains unexplored.

The aim of this study was to evaluate the antiparasitic properties of 3,6-epidioxy-1,10-bisaboladiene (EDBD), a diastereomeric mixture of endoperoxide sesquiterpenes obtained from *D. brasiliensis*, against *Schistosoma mansoni* both in vitro and in an animal model of schistosomiasis. In vitro experiments determined its effective concentration 50% (EC_50_), while in vivo studies assessed its efficacy in *S. mansoni*-infected mice. Additionally, the toxicity profile of EDBD was evaluated in mammalian cells, providing valuable insights into its safety and potential efficacy.

## 2. Results

### 2.1. Chemical Characterization of EDBD 

ESI-HRMS (Figure S1) showed a [M+H]^+^ ion peak at *m*/*z* 237.1852 (calculated for C_15_H_25_O_2_, 237.1854), confirming the molecular formula as C_15_H_24_O_2_, with four unsaturation. ^1^H NMR spectrum (Figure S2) displayed, along with other signals, two coupled doublets (*J* = 8.5 Hz) at δ 6.45 and 6.38, assigned to the olefinic hydrogens H-1 and H-2, respectively, whereas the triplet at δ 5.08 (*J* = 6.0 Hz) was attributed to H-10. The presence of four methyl groups was inferred by the occurrence of three singlets at δ 1.67 (H-12), 1.60 (H-13), and 1.37 (H-15) and one doublet at δ 0.99 (*J* = 6.4 Hz, H-14). 

The ^13^C (Figure S3) and DEPT (Figure S4) NMR spectra showed 15 signals, being four methyl, four methylene, four methine and three quaternaries, confirming the occurrence of a sesquiterpene. Among these signals, four corresponding to sp^2^ carbons were observed at δ 136.3 (C-1), 133.5 (C-2), 124.1 (C-10) and 131.8 (C-11). Additionally, two carbinolic carbons were observed at δ 79.9 (C-6) and 74.4 (C-3), suggesting the presence of an endoperoxide moiety. 

**Figure 1:**
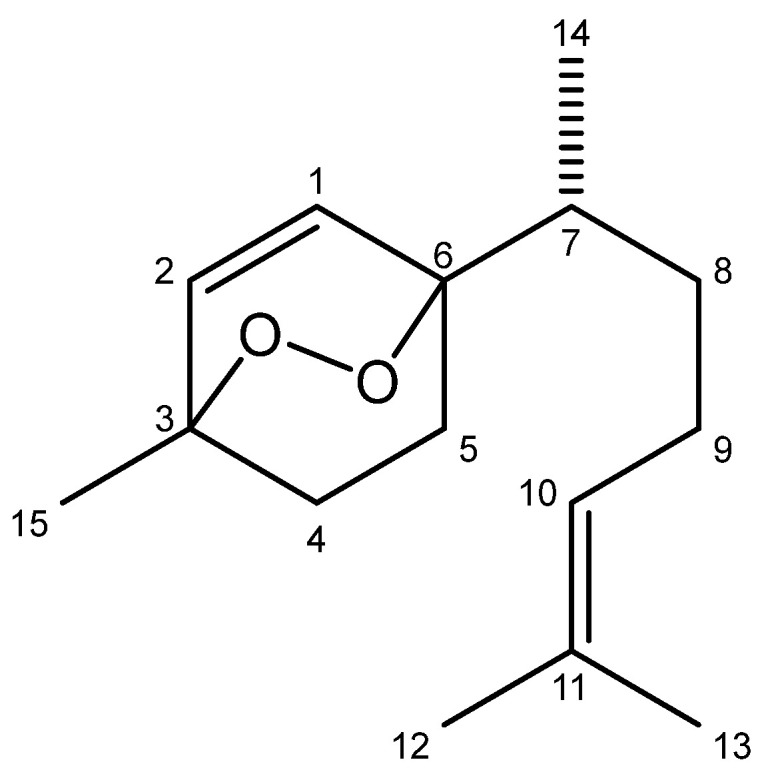
Chemical structure of EDBD (3,6-epidioxy-bisabola-1,10-diene), isolated from the hexane extract of branches of *D. brasiliensis*.

Finally, analysis of HSQC (Figure S5) and HMBC (Figure S6) spectra followed by comparison of the obtained data with those previously published [14], confirmed the chemical structure of 3,6-epidioxy-bisabola-1,10-diene—EDBD (Figure 1). 

### 2.2. EDBD Exhibited Antiparasitic Effects on S. mansoni without Toxicity to Mammalian Cells 

**Figure 2:**
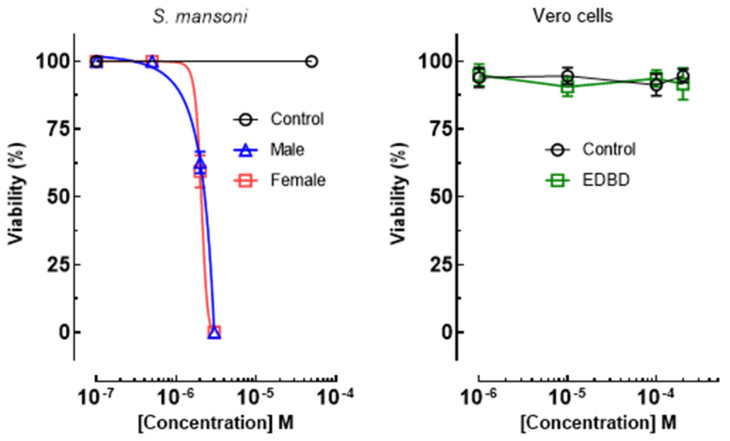
Effect of EDBD (3,6-epidioxy-bisabola-1,10-diene) on the viability of *S. mansoni* and Vero cells. Adult schistosomes and cells were incubated for 72 h in the presence of different concentrations of EDBD. RPMI 1640 medium with 0.5% DMSO, representing the highest concentration of solvent, was used as the negative control. Values are mean (±SD) from three independent experiments performed in triplicate.

The in vitro antiparasitic effect of EDBD against *S. mansoni* adult worms (both male and female) was evaluated to determine the effective concentration 50% (EC_50_). PZQ served as the positive while 0.5% DMSO, representing the highest concentration of solvent, was used as the negative control. As shown in Figure 2, parasites in the negative control group (0.5% DMSO) remained viable throughout the incubation period. In contrast, PZQ caused the mortality of all schistosomes immediately. EDBD exhibited significant antiparasitic effects at low micromolar concentrations. The EC_50_ values are summarized in Table 1. EDBD displayed marked anthelmintic activity, with EC_50_ values of 4.1 μM for both male and female adult schistosomes. These values are comparable to the positive control PZQ, which had EC_50_ values of 1.1 μM and 1.3 μM, respectively.

To evaluate toxicity, the Vero mammalian cell line was used to determine the CC_50_ and the selectivity index (SI) values of EDBD. Notably, EDBD exhibited no cytotoxicity against Vero cells (Figure 2), with CC_50_ values exceeding 200 μM, resulting in a favorable SI > 48 for adult schistosomes (Table 1), underscoring the compound’s safety profile in an animal model.

### 2.3. In Silico Evaluation 

**Figure 3:**
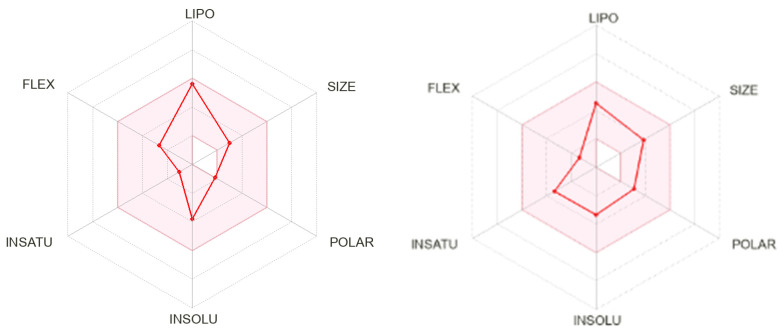
Bioavailability radar of EDBD (3,6-epidioxy-bisabola-1,10-diene—**left**) and PZQ (praziquantel—**right**).

Considering the in vitro activity of EDBD, an in silico analysis was conducted using the SwissADME platform, which investigates the pharmacokinetic and pharmacodynamic properties of molecular structures. An important tool for interpreting these data is the bioavailability radar (Figure 3), which assesses a drug’s absorption rate into the bloodstream and its biotransformation for excretion. Upon reviewing the image, it is observed that DBD exhibits properties similar to those of the positive control, PZQ.

Table 2 presents the in silico physicochemical values and ADME parameters of EDBD in comparison to PZQ. Notably, EDBD does not violate any of Lipinski’s, Ghose’s, Veber’s, Egan’s, or Muegge’s rules, indicating they have a molecular weight (MW) < 500 Da, log P_o/w_ < 5, and topological polar surface area (TPSA) < 140 Å. These values suggest high absorption and good oral availability [15], positioning them as lead-like compounds. Additionally, no alerts for PAINS were detected for either tested compound.

### 2.4. In Vivo Evaluation of EDBD—Reduction in Adult Worm Parasite Burden and a Decrease in Egg Production

**Figure 4:**
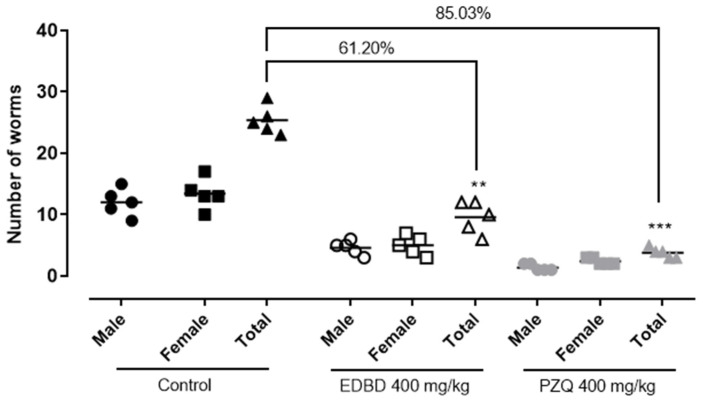
Effect of 3,6-epidioxy-bisabola-1,10-diene (EDBD) and praziquantel (PZQ) on worm burden in *S. mansoni*-infected mice. Mice were infected with *S. mansoni*, and forty-two days later, they received a single oral gavage dose of either the test compounds (400 mg/kg) or the vehicle (control). Fifty-six days post-infection, all animals were euthanized, dissected, and all schistosomes were removed, sexed, and counted. Points represent data from individual animals (n = 5 per group). Data are presented as the mean ± SD from five animals per group (n = 5). ** *p* < 0.01; *** *p* < 0.001 compared with the infected vehicle-treated control.

Based on the promising in vitro and in silico findings, in vivo testing of EDBD was conducted in *S. mansoni*-infected mice. Animals were orally administered 400 mg/kg of EDBD, the standard dose used in schistosomiasis drug discovery programs with a murine model infected with *S. mansoni* [16], and compared with infected but untreated controls. Treatment with EDBD led to a significant reduction in parasite burden by 61.20%, while the antiparasitic drug PZQ caused a worm burden reduction of 85.03% (Figure 4). 

**Figure 5:**
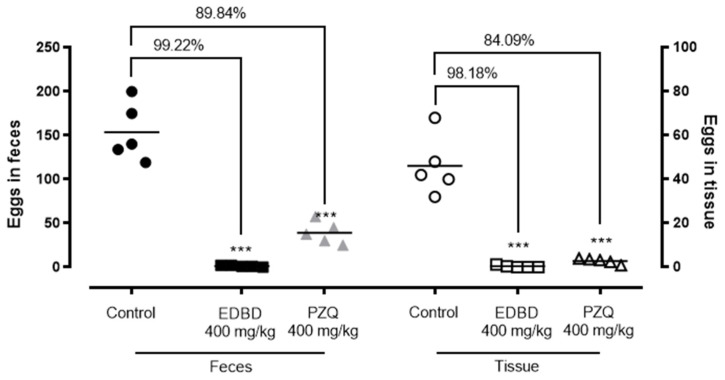
Effect of 3,6-epidioxy-bisabola-1,10-diene (EDBD) and praziquantel (PZQ) on egg burden in *S. mansoni*-infected mice. Forty-two days after infecting mice with *S. mansoni*, a single oral gavage dose (400 mg/kg) of either the test compounds or the vehicle (control) was administered. Mice were euthanized and dissected 56 days post-infection. Egg burden was assessed in both feces and intestinal tissue, with the tissue burden based on the number of immature eggs. Data points represent individual animals (n = 5 per group). The results are presented as the mean ± SD for each group (n = 5). Compared to the infected vehicle-treated control group, both compounds significantly reduced the egg burden (*** *p* < 0.001).

In addition to parasite burden reduction, treatment with EDBD resulted in a marked decrease in egg production, with reductions of 99.22% in feces and 98.18% in intestines (*p* < 0.001), surpassing the effectiveness of PZQ, which achieved reductions of 84% to 89% in egg burden (Figure 5).

## 3. Discussion

In this study, the diastereomeric mixture (1:1) of endoperoxide sesquiterpene 3,6-epidioxy-bisabola-1,10-diene (EDBD) was obtained from the hexane extract of *D. brasiliensis* branches and was characterized by NMR (¹H and ¹³C) and ESI-HRMS analysis. The biological results of this study demonstrate that EDBD exhibits significant antiparasitic activity against *S. mansoni*, a major parasitic pathogen causing schistosomiasis. The effectiveness of EDBD is underscored by its reduced EC_50_ value (4.1 μM) for both male and female adult worms, which is comparable to the commonly used antiparasitic drug PZQ. This highlights the potential of EDBD as a viable alternative or complement to existing treatments. 

The significance of the endoperoxide group is particularly notable, as literature data describe its importance for the antiparasitic activity of similar molecules such as ascaridole and artemisinin [17]. Although the reasons for sex-dependent drug sensitivity in *S. mansoni* are not fully understood, several natural products with antischistosomal properties are known to exert varying effects on male and female parasites [18]. It is possible that the targets of EDBD are present in both sexes. 

Evaluating the safety profiles of compounds is critical in the antischistosomal drug discovery process to prioritize chemical entities devoid of overt toxicity [18,19]. In this study, the in vitro assays indicated that EDBD effectively targets adult schistosomes without exhibiting cytotoxicity toward Vero mammalian cells. The high selectivity index (SI > 48) further emphasizes its safety and efficacy, distinguishing it from other compounds that may show toxicity at therapeutic doses [20]. 

The in silico analysis provided insights into the pharmacokinetic and pharmacodynamic properties of EDBD in comparison to the control PZQ. EDBD exhibited excellent compliance with several drug-likeness rules, which predict high absorption and oral bioavailability. These properties are critical for the development of oral medications [21], suggesting that EDBD is a promising candidate for further drug development.

The in vivo studies further validated the antiparasitic potential of EDBD. In *S. mansoni*-infected mice, oral administration of the compound significantly reduced the parasite burden by 61.20%. Although this reduction is substantial and demonstrates EDBD’s ability to combat schistosome infections in a living organism, PZQ showed superior efficacy, achieving an 85.03% reduction in worm burden. This higher efficacy may be attributed to the rapid action of PZQ in killing adult worms. Notably, EDBD led to an almost complete reduction in egg production, with reductions of 99.22% in feces and 98.18% in intestines, surpassing the efficacy of PZQ in this regard. Since egg production is a major factor in the pathology and transmission of schistosomiasis [4], the ability of EDBD to inhibit egg production so effectively is particularly noteworthy. 

For comparison, previous studies have reported varying levels of efficacy with different compounds, including several natural products. The lignan licarin A, at a single oral dose of 400 mg/kg, showed a 46.25% reduction in adult worms [22], while the sesquiterpene nerolidol exhibited a 70.1% reduction in adult worms and a 69.7% reduction in eggs within the intestine [23]. Notably, a single oral dose of 400 mg/kg of EDBD effectively impacted *S. mansoni*-infected mice more than these compounds reported in the literature [22,23], both in terms of adult worm reduction and egg deposition. These findings suggest that EDBD could serve as a promising prototype for schistosomiasis treatment. Additionally, they open up the possibility for future studies on the synergistic effects of these compounds and their structure–activity relationships with similar molecules, such as ascaridole. Further research could explore these aspects to optimize the antiparasitic efficacy and safety of EDBD and related compounds.

Finally, several limitations should be acknowledged in this study. Firstly, the pharmacokinetic properties of EDBD were predicted in silico, and no in vivo pharmacokinetic studies were performed. Consequently, the actual ADME profile of EDBD remains unknown, which could impact its effectiveness and safety in a clinical setting. Additionally, this study focused solely on a murine model, which may not fully capture the drug’s efficacy and safety in humans. Future research should include comprehensive pharmacokinetic evaluations and clinical trials to confirm EDBD’s potential as an effective treatment for schistosomiasis.

## 4. Materials and Methods

### 4.1. General Procedures

NMR spectra were recorded using CDCl₃ (Sigma-Aldrich, St Louis, MI, USA) as the solvent and TMS as the internal standard on a Varian INOVA spectrometer, operating at 500 MHz for ^1^H and 125 MHz for ^13^C nuclei. The ESI-HRMS spectrum was measured on a Bruker Daltonics MicroTOF QII spectrometer. For column chromatography (C.C), silica gel (230−400 mesh, Merck, Darmstadt, Germany) was used, while silica gel 60 PF254 (Merck, Darmstadt, Germany) was employed for analytical TLC separations.

### 4.2. Plant Material

Branches of *D. brasiliensis* were collected in March 2021 from the Serra do Cipó National Park, Minas Gerais State, Brazil, by Dr. Guilherme M. Antar. The collection was registered under code A4123E4 in SISGEN. A voucher specimen (SPF4105) has been archived at the Botanical Institute of São Paulo, São Paulo State, Brazil.

### 4.3. Isolation of EDBD (3,6-Epidioxy-Bisabola-1,10-Diene)

Dried branches of *D. brasiliensis* (316 g) were exhaustively extracted using hexane (10 × 500 mL) at room temperature. After evaporation of the solvent under reduced pressure, 16 g of crude extract was obtained. A portion of this extract (15 g) was subjected to chromatography on a silica gel column eluted with increasing amounts of hexane/ethyl acetate (1:0, 9:1, 8:2, 7:3, 6:4, 3:7, and 0:1), resulting in 21 fractions that were combined into five groups (A–E). Part of group C (300 mg) was further purified by silica gel column chromatography using hexane/ethyl ether (98:2, 9:2, 8:2, and 1:1) to yield 140 mg of EDBD.

*EDBD* (*3,6-epidioxy-bisabola-1,10-diene*), pale yellow oil. ^1^H NMR (500 MHz, CDCl_3_): δ 6.45 (d, *J* = 8.5 Hz, H-1), 6.38 (d, *J* = 8.5 Hz, H-2), 5.08 (t, *J* = 6.0 Hz, H-10), 2.10 (m, H-9a), 2.02 (m, H-4a), 1.94 (m, H-9b), 1.93 (m, H-5a), 1.73 (m, H-7) 1.67 (br s, H-12), 1.62 (m, H-8a), 1.60 (br s, H-13), 1.50 (m, H-4b), 1.37 (s, H-15), 1.14 (m, H-8b) 0.99 (d, *J* = 6.4 Hz, H-14); ^13^C NMR (125 MHz, CDCl_3_): δ 136.3 (C-2), 133.5 (C-1), 131.8 (C-11) 124.1 (C-10), 79.9 (C-6), 74.4 (C-3) 36.7 (C-7) 31.1 (C-8), 29.4 (C-4) 26.0 (C-9), 25.8 (C-12) 25.5 (C-5) 21.4 (C-15), 17.6 (C-13) 13.8 (C-14); ESI-HRMS *m*/*z* 237.1852 [M + H]^+^ (calculated for C_15_H_25_O_2_ 237.1854).

### 4.4. In Silico Analysis

The pharmacokinetics, drug likeness, and medicinal chemistry parameters of EDBD (3,6-epidioxy-1,10-bisaboladiene) and PZQ were evaluated in silico using the SwissADME platform (http://www.swissadme.ch/, accessed on 1 June 2024) [24]. Several parameters were evaluated: (i) Absorption, Distribution, Metabolism, and Excretion (ADME); (ii) physicochemical properties such as the number of rotatable bonds, H-bond donors, and H-bond acceptors; (iii) lipophilicity (log *p* value); (iv) pharmacokinetics aspects like gastrointestinal absorption and CYP 450 inhibition; (v) drug likeness criteria based on the Lipinski (Pfizer, Hong Kong), Veber (GlaxoSmithKline, Hong Kong), and Muegge (Bayer, Leverkusen, Germany) filters; and (vi) identification of pan-assay interference compounds (PAINS).

### 4.5. Animals, Worms and Cells

The life cycle of the helminths (Belo Horizonte [BH] strain) was sustained through passage in *Biomphalaria glabrata* snails and Swiss mice at the Research Center on Neglected Diseases, Guarulhos University, SP, Brazil. The snails and mice were kept under controlled environmental conditions (25 °C, 50% humidity) with unrestricted access to food and water. Vero cells obtained from the American Type Culture Collection (ATCC) were cultured in DMEM supplemented with 10% fetal bovine serum (FBS) and 2 mM L-glutamine [25].

### 4.6. In Vitro Antischistosomal Assay 

The in vitro anthelmintic assay followed established protocols [26]. Adult schistosomes, obtained from infected mice, were exposed to varying concentrations (0.78 to 50.0 μM) of EDBD and PZQ in RPMI 1640 culture medium supplemented with 5% heat-inactivated fetal calf serum, 100 U/mL penicillin, and 100 μg/mL streptomycin in 24-well culture plates (Sigma-Aldrich, St Louis, MI, USA). Each well contained one pair of parasites (one male and one female). The compounds were dissolved in 0.5% DMSO, and each concentration was tested in triplicate, with the experiments repeated three times for reliability.

Parasite motility was observed daily at 24, 48, and 72 h using a BEL Engineering microscope (INV 100, Monza [MB], Italy). A worm was considered deceased if it remained motionless everywhere on its body for at least 1 min, even upon stimulation with tweezers.

### 4.7. Toxicity Profile in Mammalian Cells

The cytotoxicity assessment followed established protocols [21,22]. Cells were cultured in 96-well plates using DMEM supplemented with 10% heat-inactivated fetal calf serum and 2 mM L-glutamine. After allowing 24 h for cell adhesion at 37 °C and 5% CO_2_, the 50% cytotoxic concentrations (CC_50_) of EDBD and PZQ were determined over a concentration range of 0.12–200 μM. Following a 72-h incubation period, MTT solution was added, and the plates were further incubated for 3 h. Subsequently, the absorbance was measured at 595 nm using an Epoch Microplate Spectrophotometer (BioTek Instruments, Winooski, VT, USA). The assay was performed in triplicate and repeated three times to ensure reliability. Results were calculated as a percentage relative to the control.

Selectivity indices (SI) were calculated by dividing the CC_50_ values obtained in Vero cells by those determined in *S. mansoni*. 

### 4.8. In Vivo Therapeutic Efficacy in an Animal Model of Schistosomiasis

Female mice (3 weeks old) were subcutaneously infected with 80 *S. mansoni* cercariae each. At forty-two days post-infection, corresponding to the presence of adult worms (patent infection), EDBD was administered as a single dose of 400 mg/kg to a group of five mice [27,28]. Control groups included *S. mansoni*-infected mice treated with a vehicle or PZQ at 400 mg/kg (five mice per group).

At 56 days post-infection, all experimental groups were weighed, euthanized, and dissected. Schistosomes were collected from the hepatic portal system and mesenteric veins, and their numbers were counted after sexing. Therapeutic efficacy was assessed qualitatively and quantitatively by examining the presence of immature eggs in the intestine. Additionally, the Kato–Katz method was used for quantitative fecal examination to provide further insights into the treatment outcomes [29].

### 4.9. Statistical Analysis

Data were analyzed using GraphPad Prism 8.0. CE_50_ and CC_50_ values were obtained from the dose–response curves. The Kruskal–Wallis test was used for in vivo studies, with a *p* value of <0.05 considered statistically significant.

## 5. Conclusions

In conclusion, the results of the present study demonstrated the anti-schistosomal activity of a diastereomeric mixture of the endoperoxide sesquiterpene 3,6-epidioxy-1,10-bisaboladiene (EDBD) obtained from branches of *D. brasiliensis* (Winteraceae). In vitro assays showed worm mortality with EC_50_ values of 4.1 μM, reducing the number of male and female parasites. Based on these in vitro results, EDBD underwent in vivo assays using a murine infection model, where there was a reduction in worm burden (61.20%) and in egg production in feces (99.22%) and tissues (98.18%), indicating superior efficacy of the compound compared to the positive control PZQ. Importantly, EDBD did not demonstrate cytotoxicity in mammalian cells. Therefore, EDBD represents a promising candidate for the development of new therapeutic approaches against schistosomiasis.

## Figures and Tables

**Table 1:** In vitro antischistosomal activities and toxicity of EDBD (3,6-epidioxy-bisabola-1,10-diene) and positive control PZQ (praziquantel).

Compound	*S. mansoni* Male EC_50_/μM	*S. mansoni* Female EC_50/_μM	Vero Cell CC_50/_μM	SI ^a^ (Male Worms)	SI ^a^ (Female Worms)
EDBD	4.1 ± 1.2	4.1 ± 1.2	˃200	˃48	˃48
PZQ	1.1 ± 0.7	1.3 ± 0.8	˃200	˃181	˃153

^a^ Selectivity index (SI) = CC_50_/EC_50_. Values are means (±SD) of three independent experiments performed in triplicate.

**Table 2:** In silico physicochemical and ADME parameters of EDBD (3,6-epidioxy-bisabola-1,10-diene) and positive control PZQ (praziquantel).

Parameters	EDBD	PZQ
MW	236.35 Da	312.41 Da
TPSA	18.46 Å	40.62 Å
Log P_o/w_	3.79	3.00
Log S	−3.21	−3.52
Gastroinstestinal absorption	High	High
BBB	Yes	Yes
Inhibitor CYP1A2	No	No
Inhibitor CYP2C19	Yes	Yes
Inhibitor CYP2C9	Yes	No
Inhibitor CYP2D6	No	Yes
Inhibitor CYP3A4	No	Yes
Lipinksi	Yes	Yes
Ghose	Yes	Yes
Veber	Yes	Yes
Egan	Yes	Yes
Muegge	Yes	Yes
Bioavaliability Score	0.55	0.55
Leadlikeness	Yes	Yes
PAINS	0 alert	0 alert

## Data Availability

The data that support the findings of this study are available from the corresponding author upon reasonable request.

## Supplementary Materials

The following supporting information can be downloaded at: https://www.mdpi.com/article/10.3390/antibiotics13080779/s1, Figure S1: ESI-HR spectrum (positive mode) spectrum of EDBD—3,6-epidioxy-1,10-bisaboladiene, Figure S2: ^1^H NMR spectrum of EDBD—3,6-epidioxy-1,10-bisaboladiene, Figure S3: ^13^C NMR spectrum of EDBD—3,6-epidioxy-1,10-bisaboladiene, Figure S4: DEPT NMR spectrum of EDBD—3,6-epidioxy-1,10-bisaboladiene, Figure S5: HSQC spectrum of EDBD—3,6-epidioxy-1,10-bisaboladiene, Figure S6: HMBC spectrum of EDBD—3,6-epidioxy-1,10-bisaboladiene.
