# Supplementary material for: EDBD—3,6-Epidioxy-1,10-Bisaboladiene—An Endoperoxide Sesquiterpene Obtained from Drimys brasiliensis (Winteraceae) Exhibited Potent Preclinical Efficacy against Schistosoma mansoni Infection
Source: Antibiotics (Basel). 2024 Aug 18;13(8):779. doi: 10.3390/antibiotics13080779 (PMC11350924; doi:10.3390/antibiotics13080779)
Supplement: Supplementary file 1 [file antibiotics-13-00779-s001.zip › antibiotics-3087014-supplementary.pdf]

EDBD - 3,6-epidioxy-1,10-bisaboladiene - an endoperoxide sesquiterpene isolated from *Drimys brasiliensis* (Winteraceae) exhibited potent preclinical efficacy against *Schistosoma mansoni* infection

SUPPLEMENTARY MATERIAL

Eric Umehara[1], Thainá R. Teixeira[2], Rayssa A. Cajás[2], Monique C. Amaro[2], Josué de Moraes[2,3,*], João Henrique G. Lago[1,*]

[1]Centro de Ciências Naturais e Humanas, Universidade Federal do ABC, Santo André, SP, Brazil;

ericumehara@hotmail.com (E.U.); joao.lago@ufabc.edu.br (J.H.G.L.)

[2]Centro de Pesquisas de Doenças Negligenciadas, Universidade Guarulhos, Guarulhos, SP, Brazil;

thainanpdn@gmail.com (T.R.T.); rayssacajas@gmail.com (R.A.C.); moniquecamaro2004@gmail.com (M.C.A.); moraesnpdn@gmail.com (J. de M.)

[3]Núcleo de Pesquisas em Doenças Negligenciadas, Instituto Científico e Tecnológico, Universidade Brasil, São Paulo, SP, Brazil;

moraesnpdn@gmail.com (J. de M.)

*Correspondence:

moraesnpdn@gmail.com (J. de M.) and joao.lago@ufabc.edu.br (J.H.G.L.)

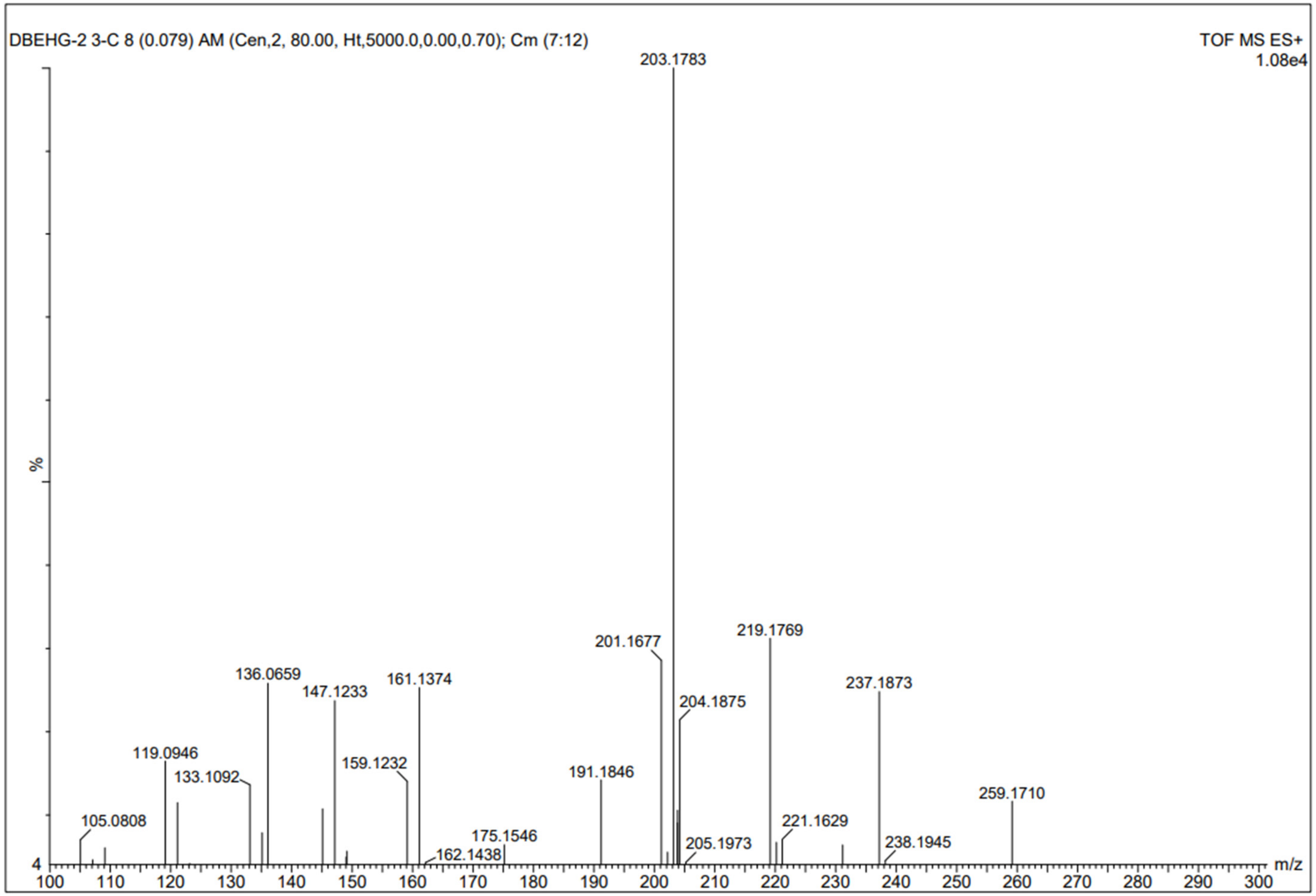

Figure S1: ESI-HR spectrum (positive mode) spectrum of EDBD - 3,6-epidioxy-1,10-bisaboladiene.

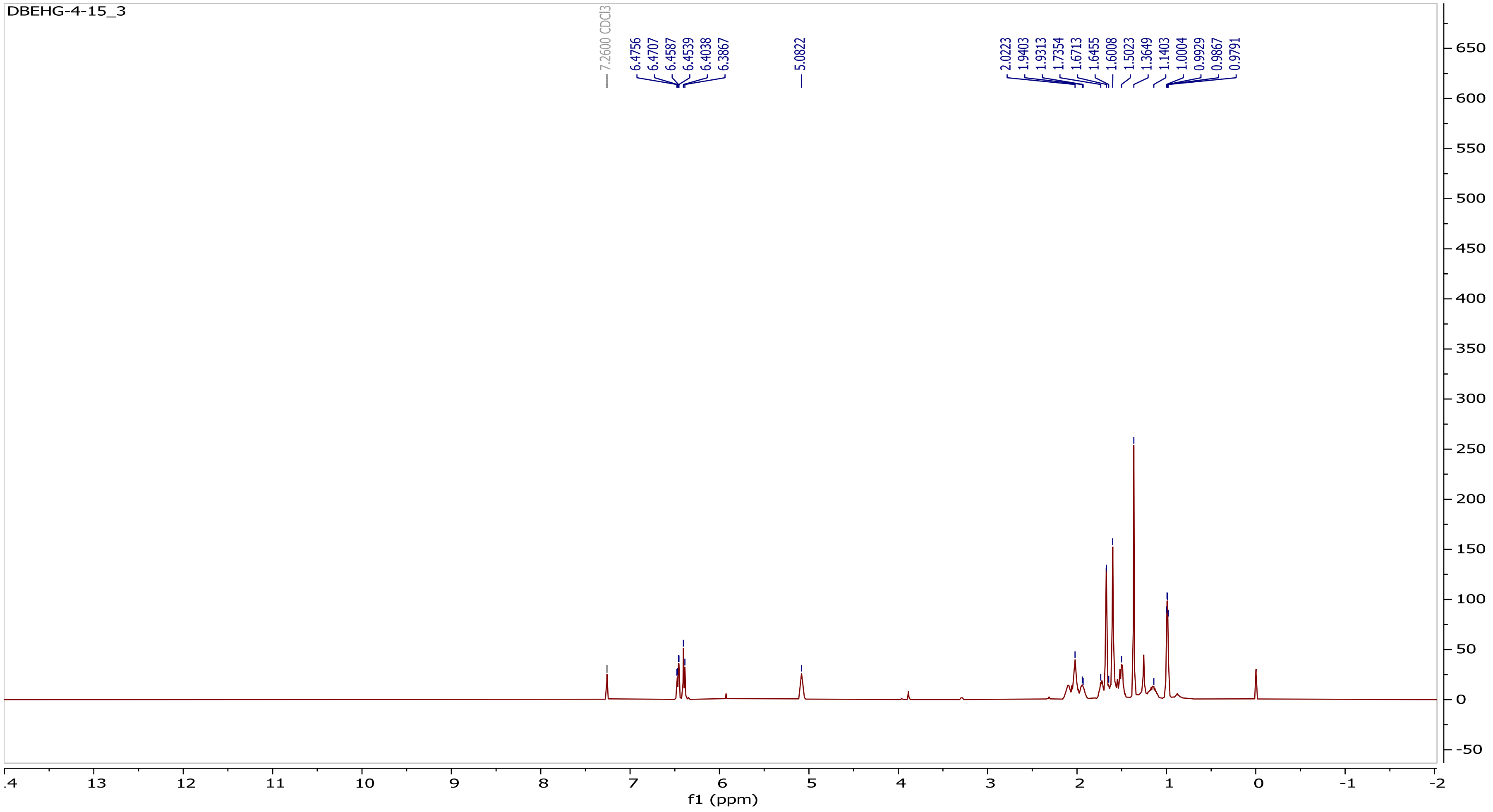

Figure S2: ^{1}H NMR spectrum of EDBD - 3,6-epidioxy-1,10-bisaboladiene.

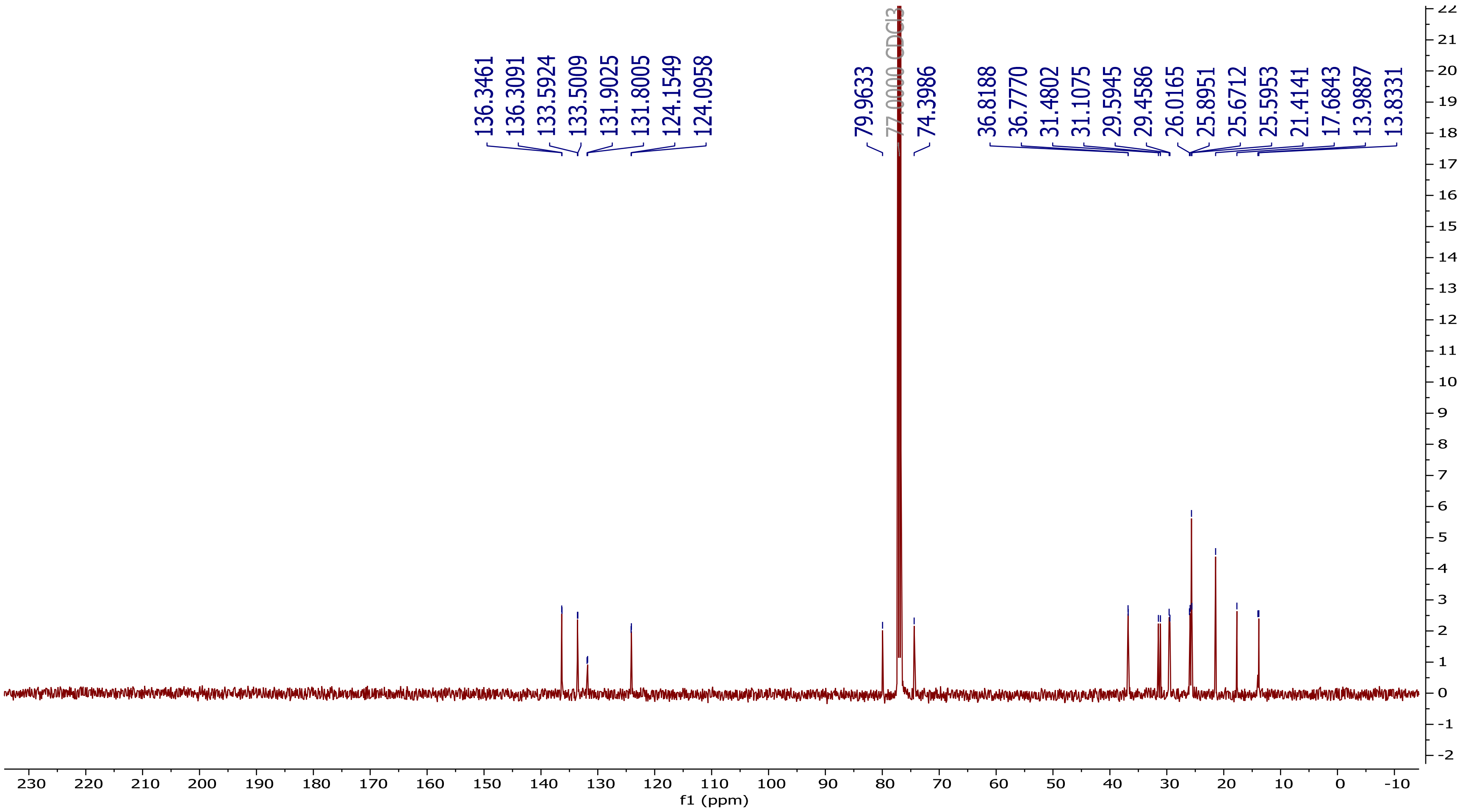

Figure S3: ^{13}C NMR spectrum of EDBD - 3,6-epidioxy-1,10-bisaboladiene.

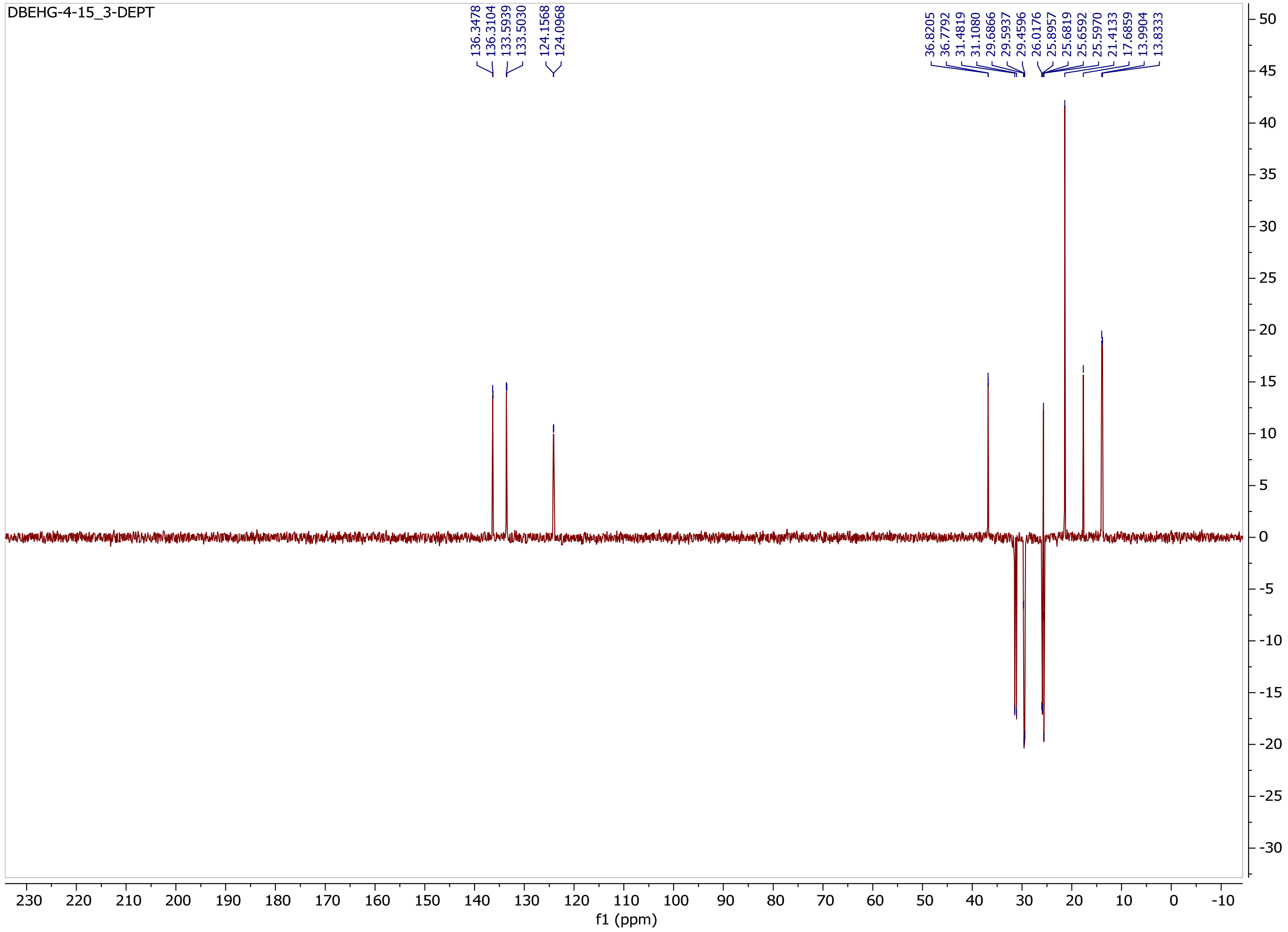

Figure S4: DEPT NMR spectrum of EDBD - 3,6-epidioxy-1,10-bisaboladiene.

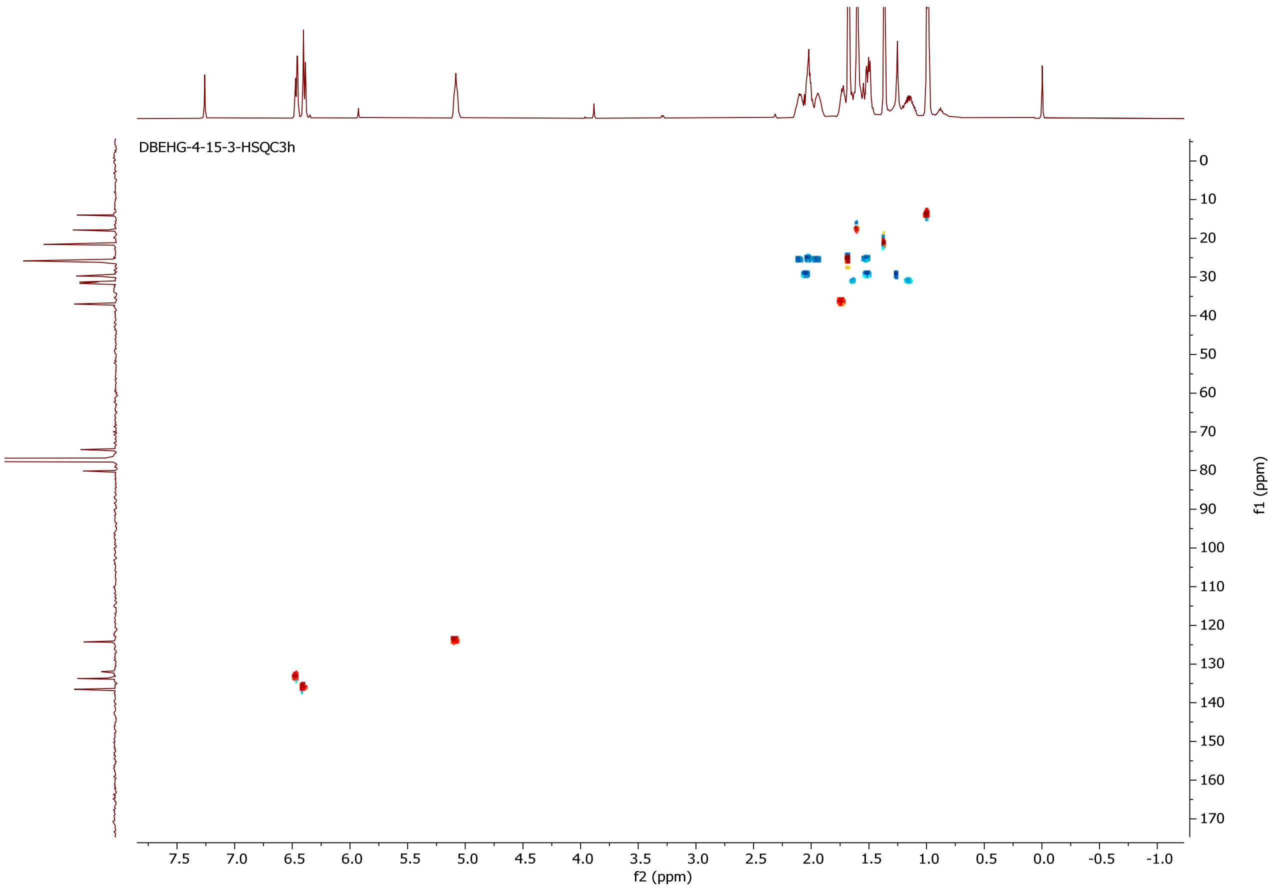

Figure S5: HSQC spectrum of EDBD - 3,6-epidioxy-1,10-bisaboladiene

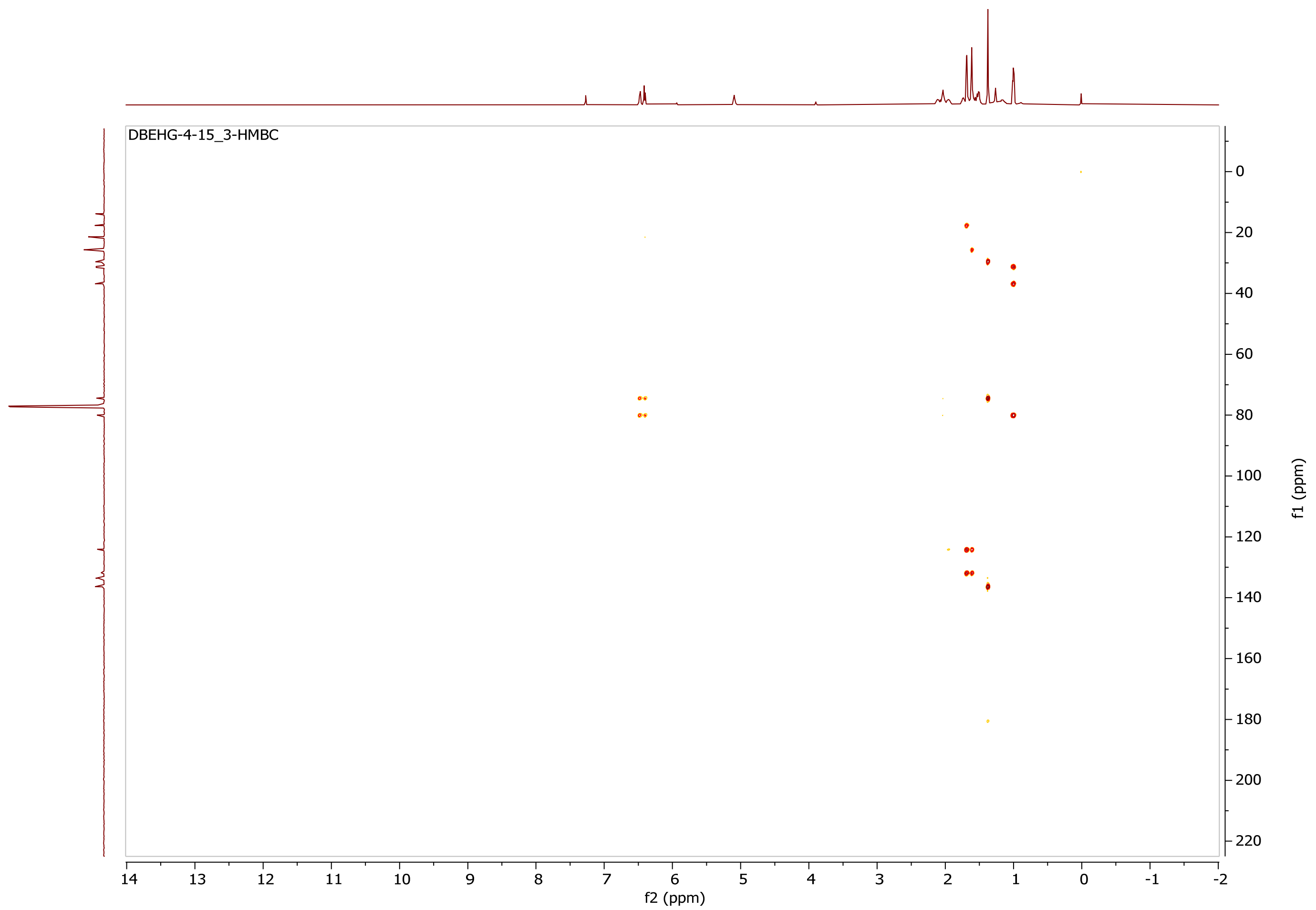

Figure S6: HMBC spectrum of EDBD - 3,6-epidioxy-1,10-bisaboladiene
